# Gamma-Mangostin, a Micronutrient of Mangosteen Fruit, Induces Apoptosis in Human Colon Cancer Cells

**DOI:** 10.3390/molecules17078010

**Published:** 2012-07-03

**Authors:** Hui-Fang Chang, Ling-Ling Yang

**Affiliations:** 1 Department of Pharmacognosy, School of Pharmacy, College of Pharmacy, and Center of e-CAM, Taipei Medical University, 250 Wusing St., Taipei 110, Taiwan; Email: beautymoon16@yahoo.com.tw; 2 Center of Translational Research on Traditional Medicine, China Medical University and Hospital, 2 Yuh-Der Road, Taichung 40447, Taiwan; 3 Graduate Institute of Clinical Medical Science, China Medical University, 91 Hsueh-Shih Road, Taichung 40402, Taiwan

**Keywords:** *Garcinia mangostana*, γ-mangostin, colorectal adenocarcinoma, antiproliferation, apoptosis, reactive oxygen species

## Abstract

Recently colorectal cancer rates have increased rapidly in Taiwan. The treatment of colorectal cancer includes surgery, radiation therapy and chemotherapy. Mangosteen (*Garcinia* *mangostana*) is a famous Asian tropical fruit. γ-Mangostin is a xanthone derivative isolated from the fruit hull. In previous studies, we found evidence of anti-inflammatory and anti-brain tumor activities in γ-mangostin. In this study, we performed further studies to assess the apoptotic effects of γ-mangostin on colorectal adenocarcinoma cells HT29. γ-Mangostin showed concentration and time-dependent cytotoxic effects on HT29 cells. Microscopic observation under Giemsa staining showed that γ-mangostin induced cellular swelling and the appearance of apoptotic bodies, characteristic of apoptosis in HT29 cells. In addition, flow cytometry analysis showed an increase of hypodiploid cells in γ-mangostin-treated HT29 cells, while enhancement of intracellular peroxide production was detected in the same γ-mangostin-treated cells by DCHDA assay and DiOC6(3) staining. In view of the above results, γ-mangostin has demonstrated anticancer activity and induces apoptosis in HT29 colorectal adenocarcinoma cells. The evidence suggests that γ-mangostin could serve as a micronutrient for colon cancer prevention and is a potential lead compound for the development of anti-colon cancer agents.

## 1. Introduction

Worldwide, every year, more than 1 million individuals will develop colorectal cancer, and the disease specific mortality rate is nearly 33% in the developed world [1]. Colon cancer has become one of the leading causes of cancer-related deaths in recent decades [2], therefore development of new effective agents or phytochemicals is necessary for the clinical treatment and prevention of colorectal adenocarcinoma. In recent years, there has been a more detailed understanding of the pathologies underlying the process of tumor growth and progression. Consequently, the design and synthesis of new drugs against deregulated signaling pathways has become possible [3,4]. Phytonutrients are naturally occurring chemical compounds or extracts in plants that are known to be beneficial to human health, and as such, chemoprevention by the use of naturally occurring substances in the food, vegetables and fruits consumed daily is becoming a promising strategy to prevent cancer [5]. 

Mangosteen (*Garcinia* *mangostana*) is imported from Thailand and cultivated at Southern Taiwan to produce a popular refreshing juicy fruit consumed in the summer. Mangosteens are small (about 4 to 8 cm in diameter) round fruits with a thick, brittle, deep purple spherical outer shell or pericarp. The edible snow white endocarp of the mangosteen is arranged in a circle of 4- to 8-wedge-shaped, segmented arils [6]. The α-, β-, and γ-mangostin xanthones are the major bioactive compounds found in the fruit hulls of mangosteen, and possess a wide range of biological activities, inhibiting cell proliferation in human brain cancer U87 MG and GBM 8401 cells [7], breast cancer by aromatase inactivation [8], showing short-term chemopreventive effects on putative preneoplastic lesions involved in rat colon carcinogenesis [9], reducing tumor growth and lymph node metastasis in a mammary cancer model [6], decreasing human immunodeficiency virus (HIV) infection [10], and even functioning as histaminergic and serotonergic receptor-blocking agents [11]. γ-Mangostin exhibited enhancement of NK cell activity in a mouse model, and it decreased the level of prostaglandin E2 (PGE2) through inhibition of cyclooxygenase (COX-2) activity and NO production [12].

For this study, we evaluated the anti-colonrectal adenocarconoma HT29 cell proliferation activity of 38 kinds of natural polyphenols isolated from Taiwanese folk medicines, and γ-mangostin was revealed as one of the strongest antiproliferative compounds. In a previous study, comparing the biological activities of α- and γ-mangostin, we had detected anti-inflammatory activity but no proliferation inhibitory ability [12,13]. In the present study, we have found γ-mangostin has potential antiproliferative activity towards colorectal adenocarcinoma cells and its mechanism(s) of action will be investigated and on the basis of molecular evidence provided by an MTT assay, morphological study, intracellular ROS levels and mitochondrial function.

## 2. Results and Discussion

### 2.1. Antiproliferation of γ-Mangostin in HT29 Cells

**Figure 1:**
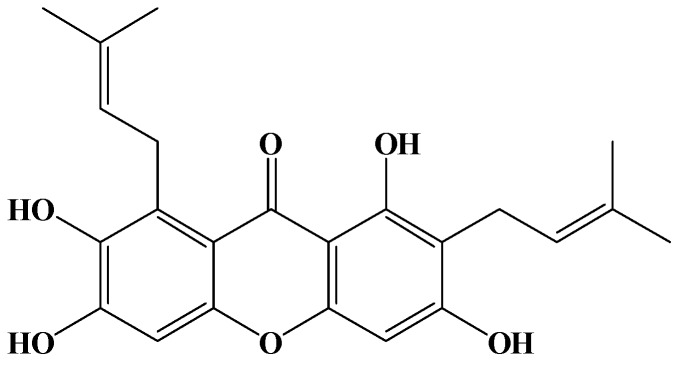
Structure of γ-mangostin.

The chemical structure of γ-mangostin is shown in Figure 1. Its effect on the viability of HT29 cells was investigated by an MTT assay (Table 1).

**Table 1:** Cytotoxicity of γ-mangostin in HT29 cells after 24 h by MTT assay.

γ-Mangostin (μM)	Cytotoxicity (%)
10	0.00 ± 4.67
20	13.16 ± 0.14
40	34.47 ± 3.13
80	80.01 ± 0.57
100	95.36 ± 3.25
200	99.90 ± 0.58

Data shown as mean ± SD, n = 3.

When cells were treated with various concentrations of γ-mangostin (10, 20, 40, 80, 100 or 200 μM) for 24 h, a significant concentration-dependent inhibition of the viability of HT29 cells was detected. Within 24 h, a significant, a rapid decrease of more than fifty percent of viable HT29 cells was recorded with IC_50_ doses (68.48 ± 6.73 μM) of γ-mangostin (Table 2). The γ-mangostin inhibited the cell proliferation in dose- and time-dependent modes.

**Table 2:** The cytotoxicity of γ-mangostin treated for different times to HT29 cells by MTT assay.

IC_50_ (µM)	Cytotoxicity (%)		
	24 h	48 h	72 h
68.48 ± 6.73	51.87 ± 4.53	69.15 ± 3.24	85.38 ± 4.75

Data shown as mean ± SD, n = 3.

In this paper, our experiment showed an extremely high IC_50_ of γ-mangostin in human colon cancer HT29 cells. In a previous report [12], the IC_50_ of γ-mangostin was 7.1 μM in human colon cancer DLD-1 cells. The colorectal cancer cells with CD133 expression exhibit enhanced tumorigenicity over CD133-negative (CD133-) cells [14]. Comparing the expression of CD133 in colorectal cancer cell lines, HT29 cell lines display relatively stronger expression of this gene than DLD-1 cells [15]. These findings suggest the anti-colon cancer activity can be compared to the CD133 gene expression capability. Therefore, HT29 cell having strong tumorigenicity than DLD-1 cells and this accounts for the showed extremely high IC_50_ of γ-mangostin in HT29 human colon cancer cells.

### 2.2. γ-Mangostin Induces the Apoptosis Process via Intracellular ROS Production

**Figure 2:**
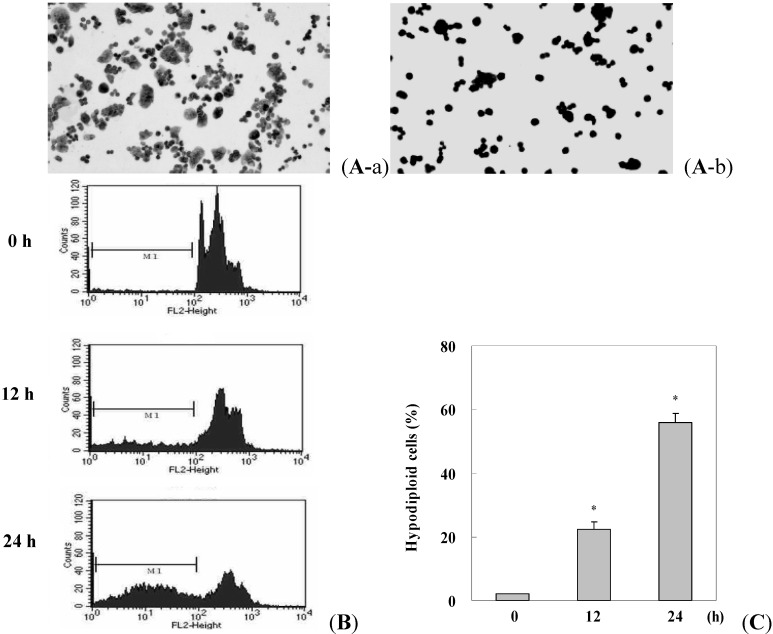
Morphological changes and hypodiploid cells inγ-mangostin treated HT29 cells. (**A**) HT29 cells were treated with (b)or without (a) γ-mangostin (80 μM) for 24 h followed by Giemsa staining, and condensed cells (b) were observed under a 100 fold zoom-in microscope; (**B**) Appearance of hypodiploid cells in γ-mangostin treated HT29 cells were shown by PI stain. Cells were treated with γ-mangostin (80 μM) for 0, 12, and 24 h, the sub-G1 (M1, hypodiploid) region was detected and quantified by flow cytometry; (**C**) Quantitative analysis of the ratio of hypodiploid cells in cells subjected to γ-mangostin (80 μM) treatment for the indicated time points. Data are presented as the mean ± SD of three independent experiments. * *p* < 0.05 and ** *p* < 0.01 indicate significant differences from the control group (CTL) as analyzed by Student’s *t*-test.

Morphological alterations in γ-mangostin-treated HT29 cells were detected under microscopic observation. After γ-mangostin treatment for 24 h, HT29 cells showed swelling and rounded morphology and the appearance of significant apoptotic bodies around the rounded cells was detected by Giemsa staining (Figure 2A). The ratio of hypodiploid cells (sub-G1 peak) in γ-mangostin treated cells significantly increased according to a flow cytometric analysis via PI staining (Figure 2B). A quantification of the sub-G1 regions (label on M1 region) relative to different time periods revealed a more sensitive apoptosis level (Figure 2C). A changeable morphology and an increase in the sub-G1 region suggest that γ-mangostin was effective as an inducer of apoptosis in HT29 colorectal adenocarcinoma cells.

**Figure 3:**
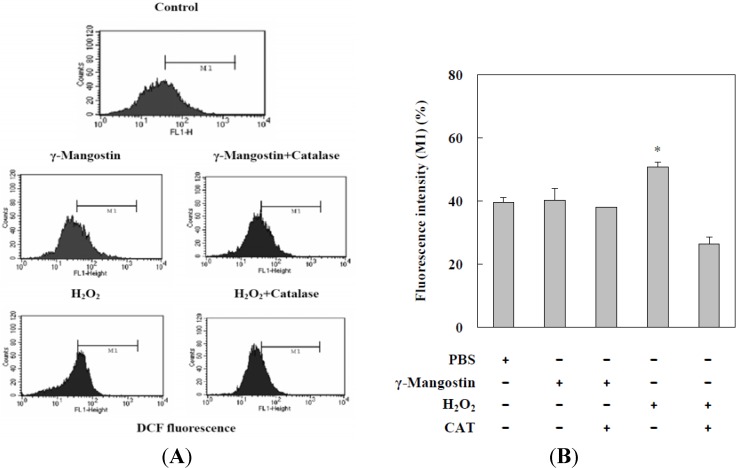
Increased intracellular peroxide levels in γ-mangostin treatedHT29 cells. (**A**) Cells were treated with γ-mangostin (80 μM) or H_2_O_2_ (1 mM) for 1 h with or without prior treatment with catalase (CAT; 400 U/mL) for 30 min. At the end of the reaction, the level of intracellular peroxide was examined by adding DCHDA for an additional 30 min followed by a flow cytometric analysis; (**B**)γ-Mangostin induced the intracellular peroxide, which was measured quantitatively through the intensity of DCFDA (DCF fluorescent intensity). Data derived from three independent experiments were calculated statistically, and results are presented as the mean ± SD. * *p* < 0.05 and ** *p* < 0.01 indicate significant differences from the control group as analyzed by Student’s *t*-test.

DCFDA has been used extensively to examine intracellular peroxide levels. Data on DCFDA showed that γ-mangostin treatment slightly increased the intracellular peroxide level, which was blocked by the addition of CAT. The H_2_O_2_ (1 mM) induced an increase in intracellular peroxide that was inhibited by the addition of CAT, and this served as a positive control. We compared γ-mangostin (80 µM) with a H_2_O_2_ (1 mM) induced system that induced intracellular peroxide production in HT29 cells, but γ-mangostin or H_2_O_2_ induced system revealed less intracellular peroxide levels which only induced about 40–50% intracellular peroxide production (Figure 3A). Quantitative data shown in Figure 3B, indicates that intracellular peroxide induced by H_2_O_2_ was reduced by the additional CAT added; however, no significant alteration in intracellular peroxide levels was detected in γ-mangostin-treated HT29 cells.

### 2.3. Cell Damage and ROS-Dependent Mitochondrial Dysfunction

**Figure 4:**
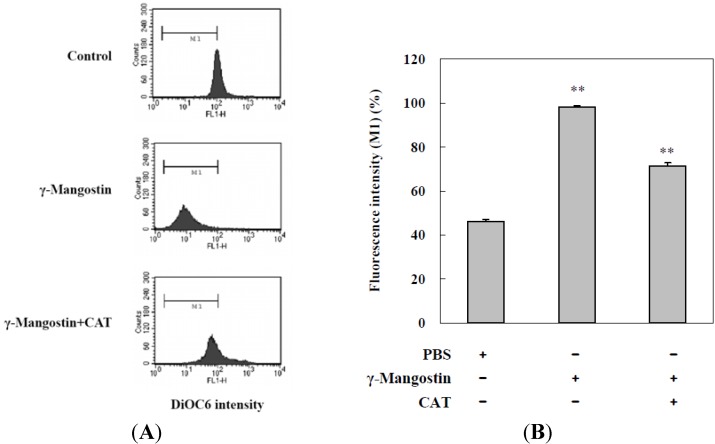
Catalase (CAT) prevented potential γ-mangostin-induced mitochondrial membrane dysfunction. (**A**) Cells were treated with γ-mangostin (80 μM) for 1 h with or without prior treatment with CAT (400 U/mL) for30 min. At the end of the incubation, the mitochondrial membrane potential was analyzed by adding DiOC_6_(3) as a fluorescent substrate on flow cytometric analysis; (**B**) To quantity data of the M1 region which is a half of control group as well as γ-mangostin induced mitochondrial dysfunction. Data derived from three independent experiments, and those results were presented as the mean ± SD. * *p* < 0.05 and ** *p* < 0.01 indicate significant differences from the control group as analyzed by Student’s *t*-test.

DiOC_6_(3) is a fluorescent dye used for detecting the function of mitochondria, and a decrease in the fluorescent intensity of DiOC_6_(3) is shown in cells with a loss of the mitochondrial membrane potential. Flow cytometry analysis data indicated that γ-mangostin treatments induced mitochondrial dysfunctions in HT29 cells, and the addition of CAT significantly prevented HT29 cells from γ-mangostin-induced mitochondrial dysfunction (Figure 4A), which was confirmed by quantitative data shown in Figure 4B that indicate that mitochondrial dysfunction induced by γ-mangostin was reduced by the addition of CAT, whereby additional CAT was able to block 30% of HT29 cells from undergoing γ-mangostin-induced mitochondrial dysfunction. In the colorectal adenocarcinoma, grade II HT29 cells, abundant intracellular reactive oxygen species (ROS) may kill it and clear the cells by mediating more than H_2_O_2_ species. HT29 cells revealed an effective tendency in sub-G1 peak production, and also showed resistance when CAT converted the function of damaged mitochondrial membranes (Figure 4B). In addition, there appeared to be a therapeutic relation to ROS induction, and the development of a strategy to improve human colorectal adenocarcinoma treatment might incorporate this feature.

## 3. Experimental

### 3.1. Chemicals

3-(4,5-Dimethylthiazol-2-yl)-2,5-diphenyltetrazolium bromide (MTT), and dimethyl sulfoxide (DMSO) were purchased from Sigma (St. Louis, MO, USA). McCoy’s 5a medium, fetal bovine serum (FBS), L-glutamine, non-essential amino acids, sodium pyruvate and penicillin-streptomycin were purchased from Gibco (Grand Island, NY, USA). All chemicals and solvents were analytical grade and purchased from Merck (Darmstadt, Germany). The propidium iodide (PI) staining solution was purchased from BD (San Jose, CA, USA). The 2'7'-dichlorofluorescein diacetate (DCFDA) fluorescent dye and 3,3'-dihexyloxacarbocyanine iodide [DiOC_6_(3)] were purchased from Invitrogen (Grand Island, NY, USA).

### 3.2. Extraction, Isolation, and Purification of γ-Mangostin

The fruit of *G. mangostana* was purchased from a fruit market in Chiayi, Taiwan. The fresh fruit hulls were separated and washed. The procedures of extraction, separation, isolation, purification, and structure determination have been described in a previous report [13]. In brief, fresh fruit hulls of *G. mangostana* were homogenized with 70% acetone. The extract was filtered and concentrated by a rotary evaporator to remove acetone, and a reddish-brown extract was obtained. The extract was dissolved in ethyl acetate (EtOAc) and filtered; the filtrate was applied to Celite 545, and then subjected to silica gel column chromatography with a gradient elution system of *n*-hexane-EtOAc. The concentrated *n*-hexane-EtOAc eluate solution was rechromatographed through a silica gel column and eluted with a CHCl_3_-MeOH gradient to afford γ-mangostin as a fine yellow powder, C_23_H_24_O_6_, with a molecular weight of 396.43. Its purity was determined by reverse-phase high performance liquid chromatography (HPLC) and was shown to exceed 98.5%. The structure of γ-mangostin, previously determined by Chen *et al.* [13] is shown in Figure 1. Test solutions of γ-mangostin (10 mM) were prepared by dissolving it in DMSO as a stock solution, whereupon it was stored at 4 °C until used. Serial dilutions of the tested solutions with culture medium were prepared immediately before each assay was performed.

### 3.3. Cell Culture

The human colon adenocarcinoma grade II cell line, HT29 was obtained from the Bioresource Collection and Research Center (BCRC) in Taiwan. HT29 cells were grown in 90% McCoy’s 5a medium supplemented with 10% fetal bovine serum. All cell cultures were incubated at 37 °C in a humidified atmosphere of 5% CO_2_. The medium was changed every 3~4 days. All cells were prepared to use at 37 °C for 24 h CO_2_ incubation after sub-cultured into new dishes by trypsinization (0.25% trypsin/0.02% EDTA).

### 3.4. Determination of Cell Viability by the MTT Assay

A MTT assay was used to measure cell viability. MTT was reduced to a purple formazan dye by mitochondrial enzymes in the process of actively respiring but not necessarily proliferating cells. Cells were seeded in a 24-well cell culture plate at a density of 3 × 10^5^ cells/mL overnight, whereupon the medium was replaced by γ-mangostin diluted with culture medium (10, 20, 40, 80, 100, and 200 μM). After 24 h drug treatment, the drug-containing medium was replaced with an equal volume (250 μL) of fresh medium containing MTT and incubated for 4 h at 37 °C. 0.04 N HCl in isopropanol (250 μL) was then added and the medium was shaken for 30 min. Finally, the optical density (OD) was detected at 600 nm and the cytotoxicity index (CI in %) was calculated according to the equation: CI (%) = [1 − (T/C)] × 100%; where T and C represent the mean OD of the treated (T) and control (C) groups. The half maximal inhibitory concentration (IC_50_) of the cells was then measured [16].

### 3.5. Morphological Study Using Fluorescence Microscopy

HT29 cells were seeded at a density of 5 × 10^5^ cells/well into 6-well plates. After 24 h of adherence, cells were treated with and without a series of concentrations of γ-mangostin (0.5–100 μM) for 24 h. Giemsa cell staining was conducted before examination by zoom-in 200 fold microscopy (OLYMPUS IX81, Core Facility Center, Office of Research and Development, TMU).

### 3.6. Apoptosis and Intracellular ROS Determination

The cells were treated with 80 μM γ-mangostin at time intervals of 0, 12, and 24 h, then washed with ice-cold phosphate-buffered saline (PBS) and fixed in 70% ethanol at −20 °C for at least 1 h. After 70% EtOH fixation, cells were washed twice, and incubated in 0.5 mL 0.5% Triton X-100/PBS at 37 °C for 30 min, and then 1 mg/mL RNaseA was added. Cells were stained with 0.5 mL of 50 μg/mL PI for 10 min. Fluorescence emitted from the propidium-DNA complex was quantitated after excitation of the fluorescent dye by FACScan flow cytometry [17]. DCHDA was used to examine intracellular peroxide levels. HT29 cells were seeded in a 24-well cell culture plate at a density of 3 × 10^5^ cells/mL and adhered for 24 h. HT29 cells were then treated with H_2_O_2_ (1 mM) and γ-mangostin (80 μM) for 1 h respectively, and then catalase (CAT, 400 U/mL) was added in order to scavenge the remaining H_2_O_2_ for half an h. Finally, the cells were dyed with DCHDA before being examined by flow cytometry [18].

### 3.7. Detection of the Mitochondrial Function

DiOC_6_(3) is a fluorescent dye used to detect the function of mitochondria with a decrease in the fluorescent intensity of DiOC_6_(3) being indicative of a loss of mitochondrial membrane potential of the cells. Cells were seeded at a density of 3 × 10^5^ cells/well in 24-well plates and underwent γ-mangostin (80 μM) treatment for 1 h. In order to evaluate the γ-mangostin induced apoptosis, the treated cells were then combined with catalase (400 U/mL) for 30 min. The cells were dyed with DiOC_6_(3) before being examined by flow cytometry [18].

### 3.8. Statistical Analysis

All values for tables and figures were presented as mean ± standard deviation (SD) for three determinations. The significance of the difference from the respective controls for each experimental test condition was assessed using Student’s *t*-test for each paired experiment. A *p* value <0.01 or <0.05 was regarded as indicating a significant difference from the control group.

## 4. Conclusions

In Thailand, the rind of mangosteen, *G. mangostana* has been used for centuries to treat trauma, diarrhea, and skin infections [19], and possesses anti-inflammatory properties [20,21,22]. Furthermore, it can promotes cell cycle arrest in prostate cancer and decreases xenograft tumor growth [23], inhibits cell proliferation in human brain cancer U87 MG and GBM 8401 cells [7], has antioxidant properties [24], anticancer activity [12], anti-inflammatory properties [13], antimetastatic activity in mouse metastatic mammary cancer models [6,25], and cardiovascular protective effects as well as strong antioxidant effects [26]. These findings provide a relevant basis for the development of xanthones as agents for cancer prevention and combination therapy with anticancer drugs.

Apoptosis lead to characteristic cell morphology changes and death, loss of the mitochondrial membrane potential. There is also a growing body of evidence indicating that ROS is able to induce apoptosis by helping to dissipate the membrane potential of mitochondria and therefore making it more permeable. Several papers have already demonstrated the mechanisms of cell cycle arrest and apoptosis by α-mangostin, which increases expression of active caspase-3 and -9 and induced mitochondria-mediated apoptosis and G1-phase arrest and S-phase suppression in the cell cycle [6], mediated by mitochondria-mediated apoptosis under control of the PI3K/Akt signaling pathway in the human breast cancer cell line MDA-MB231 [27], mediates PC-3 human prostate carcinoma cells metastasis by reduction of MMP-2, MMP-9, and u-PA expression through the suppression of the JNK1/2 signaling pathway and inhibition of NF-κB and AP-1 binding activity [28], targets mitochondria in the early phase and loss of membrane potential, resulting in induction of apoptosis in HL60 cells [29], inhibits Ca^2+^-ATPase to cause apoptosis through the mitochondorial pathway in PC12 cells [30]. It has also been shown that the antiproliferative effects of xanthones were associated with cell-cycle arrest by affecting the expression of cyclins, cdc2, and p27; G1 arrest was by α-mangostin and β-mangostin, and S arrest by γ-mangostin [31]. 

γ-Mangostin strongly inhibited human colorectal adenocarcinoma cell proliferation. Our study focused on the mechanism of γ-mangostin-induced growth inhibition in HT29 cells. We observed condensed cells and hypodiploid cells which produce intracellular ROS and mitochondrial dysfunction in the γ-mangostin treated HT29 cells. It was shown that γ-mangostin may mediate cytotoxicity via apoptosis in HT29 cells. Epidemiological studies have shown that dietary phytochemicals provide beneficial effects in relation to cancer prevention [17,32,33,34]. These findings provide a relevant basis for developing γ-mangostin as an agent for cancer prevention. We conclude that γ-mangostin is a strong lead compound candidate in the treatment of colon cancer cell growth, and may directly affect colorectal adenocarcinoma cancer susceptibility through its modulation of cell proliferation and apoptosis.
